# Feasibility of a video-delivered mental health course for primary care patients: a single-group prospective cohort study

**DOI:** 10.1186/s12875-023-01989-8

**Published:** 2023-01-23

**Authors:** Karoline Kolaas, Anne H. Berman, Erik Hedman-Lagerlöf, Anastasiya Zakrevska, Majken Epstein, Sandra af Winklerfelt Hammarberg, Erland Axelsson

**Affiliations:** 1 Center for Psychiatry Research, Department of Clinical Neuroscience, Karolinska Institutet, Norra Stationsgatan 69, SE-113 64 Stockholm, Sweden; 2 Liljeholmen Primary Health Care Center, Region Stockholm, Stockholm, Sweden; 3 Academic Primary Health Care Center, Region Stockholm, Stockholm, Sweden; 4 Division of Clinical Psychology, Department of Psychology, Uppsala University, Uppsala, Sweden; 5 Division of Psychology, Department of Clinical Neuroscience, Karolinska Institutet, Stockholm, Sweden; 6 Gustavsberg Primary Health Care Clinic, Region Stockholm, Stockholm, Sweden; 7 Division of Family Medicine and Primary Care, Department of Neurobiology, Care Sciences and Society, Karolinska Institutet, Stockholm, Sweden

**Keywords:** Anxiety disorders, Depression, Primary health care, Transdiagnostic, eHealth, Telepsychiatry, Video, Cognitive behavioral therapy

## Abstract

**Background:**

In many health care systems, primary care is tasked with offering psychological treatment for common mental disorders. Resources are often limited, which complicates widespread dissemination of traditional psychological treatments. Stepped care models where the less resource-intensive interventions are delivered first, can be employed, but often do not eliminate the need for a thorough diagnostic assessment, which can be time-consuming, has the potential to bottleneck patient intake, and can add to waiting times. Novel low-threshold formats are needed to improve access to mental health care in the primary care setting.

**Methods:**

This was a single-group prospective cohort study (*N* = 91). We assessed the feasibility of a video-delivered course as a first-line intervention for patients seeking help for mental health problems at a primary care center. The course had a transdiagnostic approach, suitable for both depression and anxiety disorders, and was based on cognitive behavioral techniques. Patients in need of psychosocial assessment, which usually entailed a four- to six-week wait, were referred by physicians or triage nurses. Study participants could start within a week, without the need for conventional diagnostic assessment, and were informed that they would be offered assessment after the course if needed. Key feasibility outcomes included participant satisfaction, attendance rates, the proportion of participants in need of additional clinical intervention after the course, and the rate of clinically significant improvement in anxiety and depression symptoms.

**Results:**

Participants scored a mean of 21.8 (SD = 4.0, 9–32, *n* = 86) on the Client Satisfaction Questionnaire-8; just below our target of 22. The mean attendance rate was 5.0/6 lectures (SD = 1.6, range: 0–6, *n* = 91). Forty-six percent (37/81) reported experiencing no need of further clinical intervention after the course. The rate of clinically significant improvement was 59% (27/46) for anxiety and 48% (22/46) for depression. No serious adverse event was reported.

**Conclusions:**

Delivering a low-threshold online video-delivered mental health course in primary care appears to be feasible. Adjustments to further improve patient satisfaction are warranted, such as offering the choice of participating online or face-to-face.

**Trial Registration:**

(ClinicalTrials.gov NCT04522713) August 21, 2020.

**Supplementary Information:**

The online version contains supplementary material available at 10.1186/s12875-023-01989-8.

## Background

Common mental health problems such as depression and the anxiety disorders are predictive of disability [1, 2], impaired quality of life [3], and considerable societal costs [4, 5]. In many healthcare systems, primary care has a responsibility to treat patients with mild to moderate mental health problems, while maintaining high quality care and short waiting times [6]. Though approximately 30–50% of patients seeking help in primary care suffer from a mental health problem [7, 8], the lack of resources often results in long waiting times. Traditional psychological treatments are rarely offered, and evidence pertaining to the effectiveness of psychological interventions when delivered in primary care is scarce [9, 10].

One way of improving access to effective psychological treatment could be to employ a stepped care model [11], meaning that patients are first offered a low threshold intervention (e.g., a self-help book), and only patients who do not respond proceed to a more resource-intensive therapy (e.g., face-to-face psychotherapy). Stepped care models incorporating variations on cognitive behavioral therapy can be both clinically effective and financially sound [12–14]. However, a limitation of many stepped care programs is that these retain a structured diagnostic interview and clinical assessment of each patient before treatment [11, 15]. In routine clinical practice, such a comprehensive assessment phase can be time-consuming, has the potential to bottleneck patient intake, and contribute to waiting times.

A more efficient use of resources in primary care settings could be to employ a transdiagnostic approach, meaning that interventions are designed to suit patients with several different psychiatric problems. Therefore, individual diagnostic assessment can be more rudimentary. Several studies have shown promising results of transdiagnostic interventions based on cognitive-behavioral principles, but they are seldom randomized controlled studies conducted in primary care [16–18]. One example of a widespread transdiagnostic intervention for mental health is the Stress Control course [19]. This is a psychoeducational course that can be delivered to a large group of participants without any previous diagnostic assessment, as an early step in a stepped care model. The main component is psychoeducation about common mental health problems including anxiety, depression, sleep disorders and stress-related problems. The Stress Control course has been used in routine care within the context of the Improving Access to Psychological Therapies (IAPT) initiative in the UK [20]. In a 2016 evaluation across five health services (*N* = 4451), around 70% of patients attended at least four lectures and were classified as course completers [21]. Of those suffering from clinical levels of anxiety, 42% were clinically significantly improved, meaning that they both reliably improved and scored below the clinical cut-off at their last attended lecture. The corresponding figure for depression symptoms was 41%. Baseline to post-intervention, standardized effect sizes were moderate to large for anxiety (*d* = 0.70) and moderate for depressive symptoms (*d* = 0.59). To our knowledge, no similar low-threshold transdiagnostic course for common mental health problems has been evaluated in a primary care setting outside of the UK. In addition, to our knowledge, no previous study has investigated the effects of a similar transdiagnostic course when delivered in an online video format. Administering the course online may have the benefit of lowering the barrier for health care seeking, and enabling patients to take part regardless of geographical distances.

The aim of this study was to assess the feasibility of a six-week transdiagnostic online video-delivered course as a first-line intervention for patients seeking help for mental health problems in a primary health care setting. In accordance with common guidelines for feasibility studies [22], we addressed the following:

1. Acceptability and process-related outcomes: Would participant satisfaction be adequate as evidenced by a mean score of at least 22 on the Client Satisfaction Questionnaire-8 [23]? Would attendance rates be adequate? We regarded these as important outcomes because we suspected that being offered to participate in a large-group video course could potentially clash with patient’s expectations of being offered traditional face-to-face psychological treatment.
2. Indication of effectiveness as a first step of a stepped care model: What percentage of participants would not be in need of any further clinical intervention after the psychoeducational course?
3. Preliminary efficacy and adverse events: Would at least 1 out of 3 participants with clinical baseline symptoms achieve a clinically significant improvement in anxiety and depression? Would there be reductions in anxiety, depression symptoms, perceived stress, disability, and risky lifestyle behaviors? Would adverse events be acceptable in light of any apparent efficacy?

## Materials and methods

### Design and setting

This was a prospective single-group cohort study designed to evaluate the feasibility of a transdiagnostic psychoeducational course based on cognitive-behavioral principles for common mental health problems in a routine primary health care setting. The project was a collaboration between Karolinska Institutet and Liljeholmen Primary Health Care Center Stockholm, Sweden. In the publicly funded Swedish health care system, primary care clinics offer affordable care for most common health problems including mild to moderate psychiatric problems, and serve as the first step or “gatekeeper” in relation to tertiary care including specialist psychiatry. Although the focus was on feasibility and acceptability outcomes, we reasoned that the study would need to be indicative of at least moderate within-group effects on anxiety and depression to motivate the study of causal effects in a randomized controlled trial. We therefore powered the study to enable pre-post mean tests of moderate effects (d = 0.45) with 80% power, given α = 0.05 and a missing data rate of 40%. Thus, we intended to recruit 68 participants with clinical self-report depression or anxiety scores. In this study, “clinical” was defined as scoring above cut-off for probable depression or a probable anxiety disorder [24, 25]. We wanted the sample to be representative of help-seeking patients in primary care and strived not to exclude patients who might benefit from the course. Therefore we also included up to 25 participants with subclinical self-report depression and anxiety scores. All results are reported in accordance with the CONSORT extension for pilot and feasibility trials [26].

### Recruitment

Participants were adults (≥ 18 years) either scoring ≥ 8 on the GAD-7 or ≥ 10 on the PHQ-9 for the clinical sample, or scoring < 8 on the GAD-7 and < 10 on the PHQ-9 for the subclinical sample [24, 27]. Applicants who reported having a severe psychiatric condition such as bipolar disorder, suicidal ideation or a psychotic disorder that would require further assessment or treatment in specialist psychiatry were excluded and referred to the appropriate healthcare services. Applicants on antidepressant medication needed to have been on a stable dose for at least six weeks. Participants were also required *not* to have planned an absence of two weeks or more during the intended duration of the course, or to be engaged in any other ongoing psychological treatment.

### Participant characteristics

The average age for the whole sample was 38 years and 63 (69%) were women. At baseline, 70 participants (77%) scored in the clinical range for depression or anxiety, and 21 (23%) had subclinical ratings and were analyzed separately. Participant characteristics are shown in Table 1.

**Table 1:** Baseline characteristics

	Clinical group (*n* = 70)	Subclinical group (*n* = 21)	Total sample (*n* = 91)
Sociodemographics			
Age in years	36.9 (11.4) 18–66	42 (13.1) 26–68	38 (12) 18–68
Female	50 (71%)	13 (62%)	63 (69%)
Married or de facto	50 (71%)	13 (62%)	63 (69%)
University education^a^	54 (77%)	18 (86%)	71 (78%)
Employment			
Working full-time	43 (61%)	12 (57%)	55 (60%)
Working part-time (< 90%)	9 (13%)	2 (10%)	11 (12%)
Unemployed	3 (4%)	0 (0%)	3 (3%)
Retired	1 (1%)	2 (10%)	3 (3%)
Student	6 (9%)	3 (14%)	9 (10%)
Other	8 (11%)	2 (10%)	10 (11%)
Lifestyle behaviors			
Daily smoking	2/71 (3%)	0/71 (0%)	2/91 (2%)
Binge drinking more than once a month or more than 9/14 (w/m) glasses/week	16/71 (22%)	1/20 (5%)	17/91 (19%
Insufficient physical activity, < 150 min/week	20/71 (28%)	2/20 (10%)	22/91 (24%)
Notably unhealthy dietary habits (diet index 0–4)	9/71 (13%)	2/20 (10%)	11/91 (12%)
At least one unhealthy lifestyle habit	36/71 (51%)	4/20 (20%)	40/91 (44%)
Clinical variables			
Previous psychological treatment	46 (66%)	11 (52%)	57 (63%)
Anxiolytic or sleep medication	13 (19%)	1 (5%)	14 (15%)
Antidepressant medication	12 (17%)	1 (5%)	13 (14%)
Psychometric questionnaires			
Anxiety symptoms (GAD-7)	12 (4.2) 3–20	4.7 (2) 0–7	10.3 (4.9) 0–20
Depression symptoms (PHQ-9)	13.4 (4.8) 4–25	6.6 (1.8) 3–9	11.8 (5.1) 3–25
Perceived stress (PSS-10)	24.3 (5.7) 14–38	17.4 (4.0) 10–27	22.7 (6.1) 10–38
Disability (WHODAS 2.0)	28.2 (15.7) 0–66.7	16.1 (10.0) 4.2–43.8	25.4 (15.4) 0–66.7
Sick leave			
Degree			
On sick-leave (25%-100%)	10 (14%)	2 (10%)	12 (13%)
Time on sick-leave			
0–1 months	8 (80%)	1 (50%)	9 (75%)
More than one month	2 (20%)	1 (50%)	3 (25%)

Estimates are n (%) or M (SD), observed range. *GAD-7* Generalized Anxiety Disorder-7 *PHQ-9* Patient Health Questionnaire-9, *PSS-10* Percieved Stress Scale-10, *WD2-12. 2.0* World Health Organization Disability Assessment Schedule 2.0, 12 items

^a^International standard classification of education 1997 (ISCED-97) level 4 or higher

### Procedure

Before the study started, the standard procedure in the primary care center was that patients seeking help for mental health issues could be referred to the psychosocial team from their general practitioner or a triage nurse. The waiting time for a first appointment was approximately 4–6 weeks. The first meeting normally started with a semi structured interview about the patient’s situation and history and a structured diagnostic interview. Thereafter the clinician and patient together decided on a primary area to focus on, typically a common mental disorder such as social phobia, depression or panic disorder. Thereafter a low threshold intervention was offered, such as guided self-help, and in case that was not sufficient, a face-to-face treatment. When this study started, all physicians and triage nurses at the primary care center were informed about the study. They were instructed to inform help-seeking patients in need of psychosocial assessment about the ongoing study as an alternative to wait for a standard diagnostic interview. Individuals who expressed interest in the study were instructed to log onto the secure study web platform where they could read more about the study, and had the option of providing informed consent after which they could complete a screening battery that also served as the baseline assessment. Applicants who had completed the screening battery underwent a brief eligibility interview with a psychologist. This took approximately 15–20 min and involved no structured diagnostic assessment. The psychologist gave practical information about the course, answered questions and emphasized that participation in the study was voluntary. Appliciants not meeting eligibility criteria or not wanting to participate after the eligibility interview, were instead referred to a regular assessment with the psychosocial team. Applicants meeting eligibility criteria and wanting to participate, were booked for the course and could start within a week. If the participant wanted to start later than two weeks after the screening, new pre-measures were administered. An individual follow-up appointment was booked approximately one week after finishing the course, if possible with the same psychologist. At this meeting, the participant and psychologist discussed whether the participant felt improved, what behavioral changes the participant had made, and which aspects, if any, that were still a problem. The participants were asked if they were in need of any further psychological counseling services. If they expressed a need for more services, further assessment was conducted. Thereafter, the participant was offered treatment according to clinical practice (e.g., cognitive behavioral therapy for common mental health disorders).

### The transdiagnostic course

**Fig. 1:**
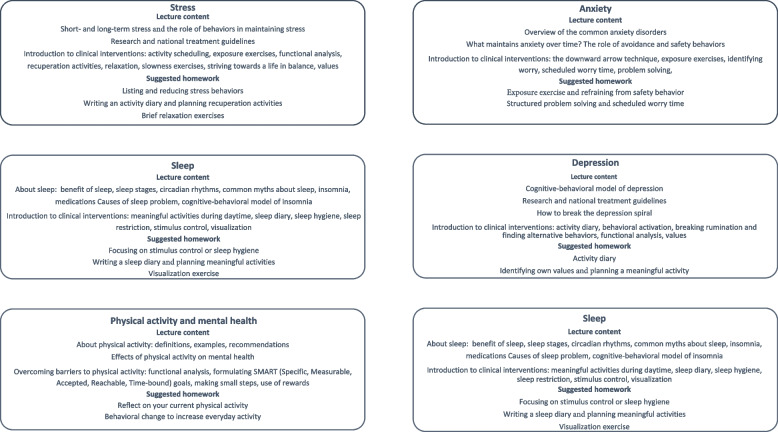
Overview of the content of each lecture

The course content was inspired by the transdiagnostic Stress Control course provided in IAPT (described earlier). The intention when designing the course was to convey evidence-based information about mental health that could inspire patients to start making behavioral and cognitive changes that would help them to improve. The course was intended to be relatively easy to deliver for a clinical psychologist trained in cognitive-behavioral theory, working in primary care. Six themes were covered: 1. Anxiety; 2. Depression; 3. Stress; 4. Sleep; 5. Physical activity and mental health, and 6. Relations and emotions. A more detailed overview is shown in Fig. 1.

Before the study started, the course was tested face-to-face in clinical practice, with extra time added at the end of each lecture for written and oral feedback from the participants. Thereafter the material was adjusted. The course was delivered live via a secure encrypted and password-protected video communication service. The participants used their own computers or smartphones to attend the lectures. The lectures made use of PowerPoint presentations, and the participants could hear and see the lecturers speaking. Participants were muted, but could unmute themselves to ask questions or to share comments with the group. There was also a chat function that allowed participants to write comments to everyone, or to the lecturers separately. Each lecture lasted for 90 min, plus short breaks of 15 min in total. One theme was given every week, and to avoid waiting times, the course was constructed for participants to be able to start with any theme and participate for six weeks running. Every lecture started with a brief introduction for new participants, a few minutes for individual evaluation of their well-being and homework done during the previous week, for those who had participated earlier. Thereafter a lecture was given on the theme of the day, consisting of both psychoeducation and advice on how to handle problems related to the theme. At the end there were suggestions for the upcoming week’s homework, and some reading suggestions on the specific theme. Each participant chose freely what to do from the homework and reading suggestions, and the individual work was not followed up. General questions and comments from the participants during the lectures were encouraged. There was no upper limit for the number of participants that could join the course, and at the most there were 16 participants in the same lecture. Two clinicians were present. One took charge of the lecturing, and the other wrote the medical records, chatted with participants who had questions and functioned as a back-up, in case the main lecturer would be ill or any other problems would arise.

The participants completed weekly questionnaires between lectures on the study web platform. If the weekly ratings were indicative of suicidal ideation, critically elevated on depression or anxiety symptoms, or adverse events, one of the psychologists phoned the participant to discuss this. If necessary, suicide risk assessment was conducted and an individual follow-up meeting could be booked by phone or video.

### Measures

#### General measurement strategy

Participants completed background information and self-report questionnaires at baseline, within two weeks before starting the course. Subsequent assessment points were weekly during the course (anxiety, depression and adverse events), after the course (including questions about satisfaction), and at follow-up three months after the six-week main phase. All questionnaires were administered online. Data were also collected as part of the eligibility check and the post-course clinical interview.

### Self-report questionnaires

Participant satisfaction was measured using the Client Satisfaction Questionnaire-8 (CSQ-8). The CSQ-8 consists of 8 questions ranging from 1–4 where 1 indicate low satisfaction and 4 indicate high satisfaction [23]. Anxiety was measured using the GAD-7 [27] and depression symptoms using the Patient Health Questionnarie-9 (PHQ-9; 24). The GAD-7 and PHQ-9 were administered in two versions: first, in their original form (assessing the past two weeks) at baseline, for the purpose of classifying participants as clinical versus subclinical; and second, in revised form to concern only the past week, administered at baseline and at all subsequent assessment points, for the purpose of evaluating change. To measure symptoms of elevated stress the 10-item Perceived Stress Scale (PSS-10; [28]) was used, and for disability the 12-item World Health Organization Disability Assessment Schedule 2 (WD2-12; [29]) was used. Lifestyle behaviors were measured using the Lifestyle Behaviors Questionnaire (LBQ) which comprises 11 items that cover tobacco use, alcohol use, physical activity and diet. A translation to English is shown in Figure S1. Adverse events were assessed using free-text items where the participant was instructed to describe up to three adverse events and rate how much they were affected by them. After finishing the course, participants were also allowed to rate how useful or helpful they had found each weekly theme, on a scale from 1 (“not at all useful/helpful”) to 10 (“very useful/helpful”).

### Statistical analysis

We conducted analyses in Stata 17. For most feasibility outcomes, no inferential statistics were employed. Clinically significant improvement was operationalized in accordance with Jacobson and Truax [30]. As pointed out above, the GAD-7 and PHQ-9 were administered in their original form at baseline, and in revised form to concern the past week at baseline and at all subsequent assessment points. For depression symptoms, only those who had a score of at least 10 on PHQ-9 at baseline could potentially be classified as clinically significant improved [24]. The corresponding cut-off for anxiety was 8 on both versions of the GAD-7 [31]. This was done to ensure that the clinical cut-off was not too liberal and also that all participants scored in the clinical range of the revised scale at baseline. The first criterion for clinically significant improvement was a reliable reduction over the six-week course as evidenced by a reduction of at least 6 points on the PHQ-9 for depression symptoms, or at least 5 points on the GAD-7 for anxiety [32]. The second criterion was a post-course score below the clinical cut-off; for missing values, this was estimated by imputation according to the last observation carried forward. We chose this method for its simplicity, and because aggregate estimates were likely to be conservative and while deviating little from the true value considering the small amount of missing data (see below). We also analyzed mean change in the GAD-7, PHQ-9, PSS-10, and WD2-12, and the potential moderating role of attended lectures, using linear mixed models. Standardized effect sizes were calculated in terms of the Cohen’s *d*, and compared with reference data from IAPT [21]. For Cohen’s *d,* effect sizes with an absolute value around 0.20 are usually regarded as small, 0.50 as moderate, and 0.80 as large [33].

## Results

### Participant flow and participation in other interventions

**Fig. 2:**
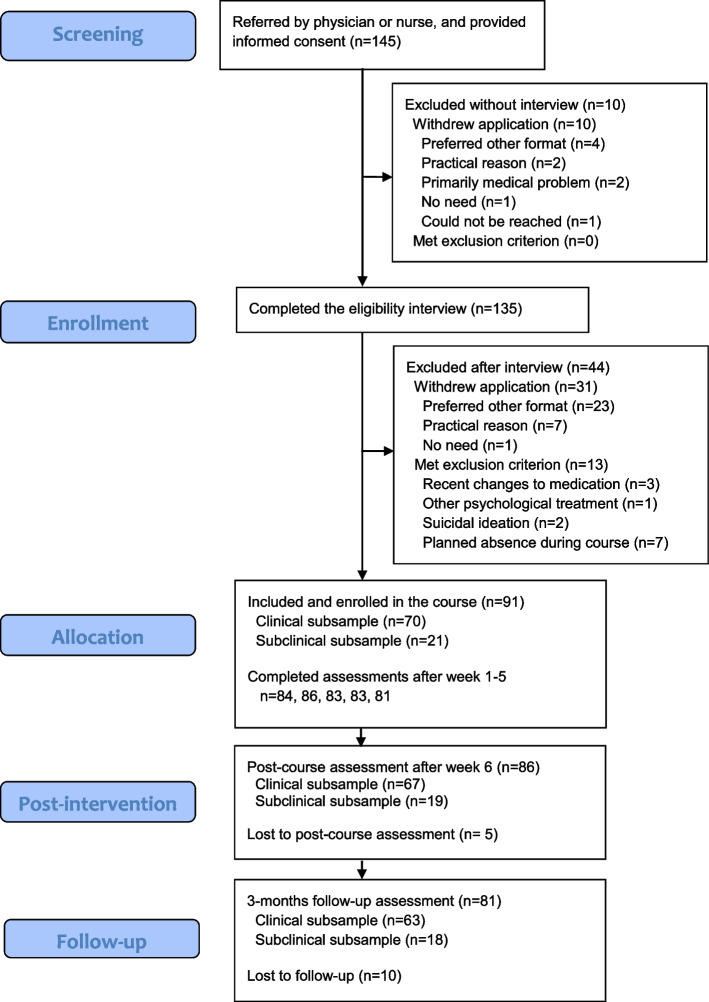
Flow chart

From September, 2020 to July 2021, ninety-one primary care patients were included in the study. The participant flow is shown in Fig. 2. Nineteen percent (17/86) reported having taken part in another clinical intervention for anxiety, stress, or depression during the course. All except one of these participants reported having attended a consultation with the general practitioner. At 3 months, 36% (29/81) had taken part in another clinical intervention since the beginning of the study; most probably often as a direct consequence of the post-course assessment. Changes to psychotropic medication were reported by 6% (5/86) after the course, and by 15% (12/81) three months later. At three months, five participants had started new medication, four had increased their dosage, three had reduced their dosage, and three had ended their medication.

### Participant satisfaction

The mean score for the whole group on the CSQ-8 was 21.8 (SD = 4.0, range: 9–32, *n* = 86), indicating that the participants were more satisfied than dissatisfied on average. For the clinical group the mean score was 21.4 (SD = 4.2, 9–32, *n* = 66) and for the subclinical group it was 23.0 (SD = 3.4, 17–31, *n* = 20). For participants who had never participated in any psychological treatment, the mean score was 22.5 (SD = 3.9, 16–32, *n* = 34), and for those who had earlier experience of psychological treatment it was 21.3 (SD 4.1, 9–29, *n* = 52). Participants rated each of the weekly course themes as useful or helpful on a scale from 1 to 10 where 1 was defined as not helpful at all and 10 as very helpful (stress: M = 6.6, SD = 2.6; anxiety: M = 6.7, SD = 2.4; depression: M = 6.5, SD = 2.4; insomnia: M = 5.9, SD = 2.8; physical activity: M = 5.8, SD = 2.8; relationships and emotions: M = 5.9, SD = 2.6).

### Attendance and therapist time

The average number of lectures attended was 5.0 of 6 (SD = 1.6, range: 0–6, *n* = 91). Eighty of 91 participants (88%) participated at least in 4 lectures. In the clinical group, the corresponding ratio was 62 of 71 (87%) and in the subclinical group it was 18 of 20 (90%). The average attendance of 5.0 lectures, each 105 min, implies that approximately 53 min of therapist time would be needed in total per participant if attending the course with a group size of 10, 35 min would be needed with a group size of 15, and 18 min with a group size of 30, not including the time needed to keep medical records.

### No further need of clinical intervention

The proportion of participants reporting no need of any additional assessment or intervention was 46% (37/81) in the sample as a whole. In the clinical group, the proportion was 42% (27/64), and in the subclinical group it was 59% (10/17).

### Clinically significant improvement

Among participants with clinical baseline levels of anxiety on the GAD-7, 63% (29/46) were reliably improved and 6% (3/46) reliably deteriorated. Clinically significant improvement, i.e., a reliable improvement to a score below the clinical cut-off, was achieved by 59% (27/46). Among participants with clinical baseline levels of depression on the PHQ-9, 52% (24/46) were reliably improved, no participant reliably deteriorated, and clinically significant improvement was achieved by 48% (22/46).

### Change in symptoms and disability

**Fig. 3:**
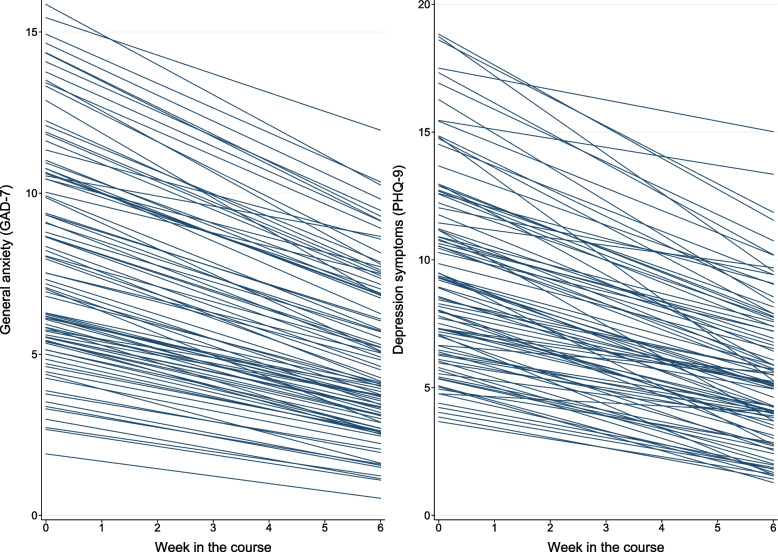
Spaghetti plots of fitted regression lines illustrating change in general anxiety (GAD-7) and depression symptoms (PHQ-9) during the transdiagnostic video-delivered course

The participants’ individual change trajectories in anxiety and depression symtoms are illustrated in Fig. 3. In the clinical group, there were significant and moderate to large average reductions in anxiety and depression (GAD-7: *d* = 0.69; PHQ-9: d = 0.75), a moderate reduction in perceived stress (PSS-10: *d* = 0.57), and a small reduction in disability (WD2-12: *d* = 0.32). Effects were sustained from the end of the course to the 3-months follow-up (GAD-7: *d* = 0.19; PHQ-9: d = 0.15). Modelled average change in symptoms of anxiety, depression, perceived stress, and disability is shown in Table S1 and changes in lifestyle behaviors are tabulated in Table S2.

### Adverse events

Seven participants (7/86 = 8%) reported having experienced one adverse event, and one participant (1/86 = 1%) reported having experienced two, during the course. The themes for these events varied. For 5 events (56%), participants mentioned a particular lecturing theme (2 sleep, 1 anxiety, 1 depression, 1 relationships/emotions). Three events (33%) consisted primarily of a temporary increase in anxiety or stress. The mean rating of distress during the incident was 1.6 (SD = 0.5, range: 1–2) on a scale from 0 (“did not affect me at all”) to 4 (“affected me very negatively”). Mean distress at the post-course assessment was 0.6 (SD = 0.7, 0–2). No serious adverse event requiring immediate care or hospitalization was reported.

## Discussion

This study evaluated the feasibility of a six-week transdiagnostic video-delivered course as a first-line intervention for patients with mild to moderate common mental health problems in a primary health care setting. For the sample as a whole, the mean overall participant satisfaction score was slightly below our preregistered target of 22 on the CSQ-8, though the mean attendance rate of 5 of 6 sessions was high. Despite the lack of a lengthy diagnostic procedure, almost half the sample had no need of additional clinical intervention. The clinically significant improvement rates of 59% in anxiety and 48% for depression clearly surpassed our preregistered target of 1/3, and there was also no indication of serious adverse events. All in all, the outcome of this feasibility study was promising, though participant satisfaction was only just about acceptable and therefore warrants attempts at improvement as discussed below.

### Comparison to previous studies

Our results indicate that the evaluated course could be suitable as a first step in a stepped care model, and may be compared to the reference study that evaluated a similar course in English primary care [21]. The completion rate was 88% in our study as compared to 70% in the reference study. The rate of clinically significant improvement in anxiety was 59% as compared to 42% in the reference study, and the corresponding figure for depression was 48% as compared with 41% in the reference study. Standardized within-group effects for the clinical group were 0.75 for anxiety and 0.69 for depression, as compared with 0.70 and 0.59 in the reference study. All in all, our results were on par with the results of a study evaluating a similar intervention widely used in clinical practice in British primary care.

### Strengths and limitations

A strength was that the course was piloted in a primary health care center, by psychologists working in the clinic with patients seeking help within the regular patient flow. This speaks for the generalizability of our findings, although it should be emphasized that we see the tested intervention as suitable only in the setting where gold standard treatments cannot be swiftly provided. The proportion of missing data was low, which increased precision. The primary limitation was the lack of a randomization and a control group. As pointed out above, UK reference data indicates that within-group effects were about the same as in the widely implemented transdiagnostic Stress Control course [21], and the 48% and 59% rates of clinically significant improvement seen here for depression and anxiety are similar to those typically reported for more conventional treatments in primary care [34, 35]. We therefore deem it unlikely that improvement was merely due to regression towards the mean, spontaneous remission, or non-specific effects such as the expectation of improvement or the attention of a clinician. This said, it is necessary to evaluate the course further in a randomized controlled trial to draw firm conclusions regarding causality. Educational attainment in this sample was high, highlighting the need to evaluate the course further in lower socioeconomic strata. It is also a limitation that a systematic qualitative component of patient satisfaction is missing, which could have given valuable information about the sub optimal ratings. Another limitation is that information was not available on the total number of patients eligible for the study. Physicians and nurses were instructed to inform all patients who would otherwise be booked for a psychological assessment (treatment as usual), about the study. However, in the busy clinical setting, it was not possible to keep track of the total number of patients that were informed about the study. Last, to make the course participants feel that they had a personal contact with someone, they usually saw the same psychologist at the follow-up consultation as at the eligibility interview which might have increased the risk that participants reported beneficial outcomes. On the other hand, self-reported depression and anxiety outcomes were largely similar to the results derived from the follow-up interview in that approximately one in two participants appeared to have benefited from the course in a clinically relevant manner.

### Potential avenues for improving the course

Even though participant satisfaction was close to our preregistered target, it was slightly lower than ideal. The patients were offered a course instead of an individual contact which is standard. This was probably not what they had expected and might have affected their patient satisfaction. We conclude that it seems important to address patient satisfaction when planning future studies and in clinical practice, both in terms of communicating what to expect and in adjusting the course to patient needs. Comments in the evaluation forms indicated that patients missed having more individualized communication with a clinican. One possibility could be to offer text-based online communication with a clinician between lectures, or the option of booking a short individual video session. This might be offered only to patients with clinical symptoms at baseline and those who have previously gone through psychological treatment earlier, as these participants were slightly less satisfied with the course. It could give participants a possibility to get individualized support, e.g. help with homework assessment or to discuss or clarify questions about the theme of the week. Another potential way of adapting the course to the individual participant needs could be to evaluate it in other formats – for example face-to-face or internet-delivered – and let the patients choose what format suits them best. The time point of filling out the questionnaire could also be of importance. In this study it was only the course satisfaction that was addressed, being a part of a stepped care model. It would be of interest in future studies to administer the CSQ-8 after the course, and again after individualized treatment for those in need, to get information about satisfaction of the whole stepped-care model. It could also be of interest with in-depth interviews of participants, to get more qualitative information about patient satisfaction.

## Conclusion

Our results support the feasibility of this six-week online video-delivered transdiagnostic cognitive-behavioral course as a first-step intervention for patients seeking help for mental health problems in primary care. With minor course revisions targeting patient satisfaction it would be of interest to evaluate the course further. Finding efficient ways of delivering low-threshold interventions that can help a large proportion of help-seeking patients in primary care could also contribute to increased access to first-line psychological treatments. This would elevate the quality of mental health primary care and save patients from unnecessary, drawn-out suffering. It is necessary to evaluate the course further in a randomized controlled trial to draw firm conclusions regarding causality. 

## Supplementary Information

**Additional file 1.**

**Additional file 2: Table S1.** Efficacy outcomes based on linear mixed models.

**Additional file 3: Table S2. **Lifestyle behaviors.

## Data Availability

The dataset supporting the conclusions in this study is not publicly available, but can be requested from the corresponding author. The scripts used for the statistical analysis are available from the corresponding author on reasonable request.

## Abbreviations

CSQ-8: Client Satisfaction Questionnaire-8
IAPT: Improving Access to Psychological Therapies
LBO: Lifestyle Behaviors Questionnaire
PHQ-9: Patient Health Questionnarie-9
PSS-10: 10-Item Perceived Stress Scale
WD2-12: 12-Item World Health Organization Disability Assessment Schedule 2
