# Supplementary material for: Feasibility of a video-delivered mental health course for primary care patients: a single-group prospective cohort study
Source: BMC Prim Care. 2023 Jan 23;24:28. doi: 10.1186/s12875-023-01989-8 (PMC9869530; doi:10.1186/s12875-023-01989-8)
Supplement: Supplementary file 1 — Additional file 1. [file 12875_2023_1989_MOESM1_ESM.docx]

Supplement 1.

**Lifestyle behavior questionnaire (LBQ)**

**Tobacco consumption**

*Mark one alternative for each question – your usual behavior*

1. **My smoking habits - tobacco**
   - I have never been a smoker
   - I have been a smoker but have quit:

date of smoking cessation ………….

- - I quit smoking more than 6 months ago
  - I smoke, but not daily
  - I smoke daily… cigarettes/day
  - I smoke tobacco other than cigarettes daily

1. **My snuff habits – snuff boxes**
   - I have never used snuff regularly
   - I have used snuff regularly but have quit:

date of snuffing cessation ………….

- - I quit snuffing more than 6 months ago
  - I use snuff, but not daily.
  - I use snuff daily… boxes/week

2/3

**Alcohol Habits**

Definition of standard drink: one ”standard drink” means 50 cl medium-strong beer, 33 cl strong beer, 12-14cl white or red wine, 8 cl fortified wine, 4 cl spirits for instance whiskey

1. **How many standard drinks do you drink in a typical week?**

Drink ….. standard drinks per week

1. **How often do you drink 5 standard drinks or more on one occasion?**

.…. times per month

**Physical activity**

1. **How much time do you spend in a typical week on physical exercise that makes you short of breath, such as running, gymnastics, ball sports?**

….. minutes per week

1. **How much time do you spend in a typical week on everyday exercise, for example walking, cycling, gardening? Add up all time (at least 10 minutes at a time)**

….. minutes per week

**Eating habits**

*Mark one option for each question – what you usually do!*

1. **How often do you eat vegetables and/or root vegetables (fresh, frozen or cooked)?**
   - Twice per day or more often
   - Once per day
   - A few times a week
   - Once a week or less often
2. **How often do you eat fruit and/or berries (fresh, frozen, canned, juice, etc.)?**
   - Twice per day or more often
   - Once per day
   - A few times a week
   - Once a week or less often
3. **How often do you eat fish or seafood as a main course?**
   - Three times a week or more often
   - Twice a week
   - Once a week
   - A few times a month or less often
4. **How often do you eat pastry, chocolate/sweets, or soft drinks/juice?**
   - Twice per day or more often
   - Once per day
   - A few times a week
   - Once a week or less often
5. **How often do you eat breakfast**
   - Daily
   - Almost every day
   - A few times a week
   - Once a week or more
