# Supplementary material for: Feasibility of a video-delivered mental health course for primary care patients: a single-group prospective cohort study
Source: BMC Prim Care. 2023 Jan 23;24:28. doi: 10.1186/s12875-023-01989-8 (PMC9869530; doi:10.1186/s12875-023-01989-8)
Supplement: Supplementary file 2 — Additional file 2: Table S1. Efficacy outcomes based on linear mixed models. [file 12875_2023_1989_MOESM2_ESM.docx]

**Table S1.**

*Efficacy outcomes based on linear mixed models*

|  | Marginal mean | | | Change Pre-Post | | | Change Post-3MFU | | |
| --- | --- | --- | --- | --- | --- | --- | --- | --- | --- |
|  | Pre | Post | 3MFU | b (95% CI) | P | Cohen’s *d* | est (95% CI) | P | Cohen’s *d* |
| Clinical (n = 70) |  |  |  |  |  |  |  |  |  |
| Anxiety (GAD-7) | 9.4 | 5.6 | 4.7 | -3.8 (-2.6, -5.0) | <0.001 | 0.69 | -0.8 (-1.7, 0.0) | 0.063 | 0.19 |
| Depression (PHQ-9) | 10.8 | 6.0 | 5.3 | -4.8 (-3.4, -6.1) | <0.001 | 0.75 | -0.8 (-1.7, 0.2) | 0.120 | 0.15 |
| Stress (PSS-10) | 24.3 | 21.1 | NA | -3.3 (-1.9, -4.6) | <0.001 | 0.57 | NA | NA | NA |
| Disability (WD2-12) | 28.2 | 24.1 | NA | -4.1 (-1.1 , -7.1) | 0.008 | 0.32 | NA | NA | NA |
|  |  |  |  |  |  |  |  |  |  |
| Subclinical (n = 21) |  |  |  |  |  |  |  |  |  |
| Anxiety (GAD-7) | 4.1 | 2.8 | 2.1 | -1.2 (-0.6, -2.3) | 0.025 | 0.47 | -0.8 (-1.8, 0.2) | 0.118 | 0.30 |
| Depression (PHQ-9) | 5.5 | 4.0 | 2.9 | -1.5 (-1.5, -2.8) | 0.030 | 0.63 | -1.1 (-2.3, 0.1) | 0.065 | 0.59 |
| Stress (PSS-10) | 17.4 | 15.9 | NA | -1.5 (1.2, -4.2) | 0.277 | 0.24 | NA | NA | NA |
| Disability (WD2-12) | 16.1 | 16.5 | NA | 0.4 (5.1, -4.3) | 0.866 | -0.04 | NA | NA | NA |

*Note*. The participants were classified as belonging to the clinical subgroup if they scored over the cut-off on either anxiety or depression, but it was not necessary to score above both. The GAD-7 and PHQ-9 were administered on a weekly basis over the 6-week course, and participants rated their symptoms pertaining to the past week. The PSS-10 and WD2-12 were administered at pre- and post-treatment only. Note that whereas a reduction in scale points corresponds to a negative coefficient (b) or negative summed slope (est), this also corresponds to a positive standardized effect size (d). Conversely, an increase in scale points corresponds to a positive coefficient (b) or positive summed slope (est), and a negative standardized effect size (d). 3MFU=3-month follow-up. NA=not applicable. PHQ-9=Patient health questionnaire 9. Post=post-treatment assessment. Pre=pre-treatment assessment. PSS-10=10-item Perceived stress scale. WD2-12=12-item World health organization disability assessment schedule 2.
