# Supplementary material for: Feasibility of a video-delivered mental health course for primary care patients: a single-group prospective cohort study
Source: BMC Prim Care. 2023 Jan 23;24:28. doi: 10.1186/s12875-023-01989-8 (PMC9869530; doi:10.1186/s12875-023-01989-8)
Supplement: Supplementary file 3 — Additional file 3: Table S2. Lifestyle behaviors. [file 12875_2023_1989_MOESM3_ESM.docx]

**Table S2.**

*Lifestyle behaviors*

| Lifestyle habit | Clinical subsample | |  | Subclinical subsample | |  | Total sample | |
| --- | --- | --- | --- | --- | --- | --- | --- | --- |
|  | Baseline | Post |  | Baseline | Post |  | Baseline | Post |
| At least one unhealthy lifestyle behavior | 51%  (36/71) | 48%  (32/67) |  | 20%  (4/20) | 21%  (4/19) |  | 44%  (40/91) | 42%  (36/86) |
| Daily smoking | 3%  (2/71) | 3.0%  (2/67) |  | 0%  (0/71) | 0%  (0/19) |  | 2%  (2/91) | 2%  (2/86) |
| Binge drinking more than once a month OR more than 9/14 (w/m)glasses/week | 22%  (16/71) | 22%  (14/67) |  | 5%  (1/20) | 5%  (1/19) |  | 19%  (17/91) | 17%  (15/86) |
| insufficient physical activity, less than 150 minutes/week | 28%  20/71 | 24%  (16/67 |  | 10%  2/20 | 16%  (3/19) |  | 24%  (22/91) | 26%  (22/86) |
| Notably unhealthy dietary habits (diet index 0-4) | 13%  9/71 | 21%  14/67 |  | 10%  2/20 | 5%  1/19 |  | 12%  11/91 | 17%  15/86 |

*Note*. Risky lifestyle behavior was defined as daily smoking, binge drinking of 4/5 (women/men) drinks at one occasion more than once a month and/or more than 9/14 (women/men) standard glasses of alcohol weekly, insufficient physical activity (<150 minutes/week) or significantly unhealthy diet habits (a score of 0-4 on a diet index in the questionnaire, ranging from 0-12).
